# Executive dysfunctions in schizophrenia measured using a virtual reality task - Jansari assessment of Executive Functions (JEF©)

**DOI:** 10.1192/j.eurpsy.2023.596

**Published:** 2023-07-19

**Authors:** E. Tyburski, A. Bober, M. Mak, E. Karabanowicz, P. Podwalski, J. Samochowiec, A. Michalczyk, L. Sagan, S. T. Mueller, E. Zawadzka, M. Folkierska, A. Jansari

**Affiliations:** ^1^Department of Health Psychology, Pomeranian Medical University in Szczecin; ^2^ Institute of Psychology, University of Szczecin; ^3^Department of Psychiatry; ^4^Department of Neurosurgery, Pomeranian Medical University in Szczecin, Szczecin, Poland; ^5^Department of Cognitive and Learning Sciences, Michigan Technological University, Houghton, United States; ^6^Department of Clinical Psychology and Neuropsychology, Maria Curie-Skłodowska University, Lublin; ^7^Faculty of Psychology, University of Warsaw, Warsaw, Poland; ^8^Department of Psychology, Goldsmiths, University of London, London, United Kingdom

## Abstract

**Introduction:**

Impairments in executive functions are often observed in schizophrenia. However, previous studies using standard tests show inconclusive and conflicting findings.

**Objectives:**

The main objective of this study was to compare the performance of schizophrenia patients and healthy controls on classical tasks and a non-immersive virtual reality task, Jansari assessment of Executive Functions (JEF^©^)

**Methods:**

A total of 71 schizophrenia patients and 80 healthy controls took part in the study. Executive functions were assessed with JEF^©^ and the following classical tasks: Color Trail Test (CTT), Stroop Color World Test (SCWT), Ruff Figural Fluency Test (RFFT), and computerized tasks from the PEBL battery: Berg Card Sorting Test (BCST), Tower of London (TOL), and Go/No Go task (GNG). The Positive and Negative Syndrome Scale (PANSS) was used to assess psychopathological symptoms.

**Results:**

Compared to healthy controls, schizophrenia patients scored lower on most of JEF^©^ indices i.e., prioritization, selective-thinking, creative-thinking, adaptive-thinking, multi-tasking, time-based prospective memory, event-based prospective memory, and action-based prospective memory (*p* < 0.001). Moreover, schizophrenia patients performed poorer on all traditional tasks (*p* < 0.001), except the GNG task.

**Conclusions:**

Schizophrenia patients were demonstrated to manifest deficits in executive functions as measured by traditional tests, such as concept formation, problem-solving, cognitive flexibility, planning or cognitive inhibition, and the executive functions measured by the JEF^©^ i.e., those that are used and observed in everyday situations such as working in an office.

This research was funded by the National Science Centre in Poland, grant number 2020/04/X/HS6/01920.

**Disclosure of Interest:**

None Declared

